# Research priorities in healthcare of persons experiencing homelessness: outcomes of a national multi-disciplinary stakeholder discussion in the United Kingdom

**DOI:** 10.1186/s12939-020-01206-3

**Published:** 2020-06-05

**Authors:** Parbir Jagpal, Karen Saunders, Gunveer Plahe, Sean Russell, Nigel Barnes, Richard Lowrie, Vibhu Paudyal

**Affiliations:** 1 School of Pharmacy, College of Medical and Dental Sciences, University of Birmingham, Edgbaston, Birmingham, B15 2TT UK; 2 Public Health England, West Midlands, Birmingham, UK; 3 West Midlands Combined Authority, Birmingham, UK; 4 Birmingham and Solihull Mental Health NHS Foundation Trust, Birmingham, UK; 5 NHS Greater Glasgow and Clyde, Glasgow, UK

**Keywords:** Homelessness, Health inequality, Research priorities

## Abstract

**Background:**

Persons experiencing homelessness (PEH) face up to twelve times higher mortality rates compared to the general population. There is a need to develop, evaluate and implement novel interventions to minimise such inequalities. This paper aims to present outcomes of a national stakeholder engagement event that was conducted to discuss research priorities around healthcare of PEH in the United Kingdom (UK).

**Main body:**

A national stakeholder event was organised in Birmingham, UK. This workshop aimed to engage diverse stakeholders from a variety of background including representations from clinical practice, substance misuse, anti-slavery network, public health practice, local authority, homelessness charities, drugs and alcohol services, Public Health England and academia.

A total of five key priority areas for research were identified which included: a) interventions to improve access to health services and preventative services; b) interventions to prevent drug and alcohol related deaths; c) improving existing services through quality improvement; d) identifying PEH’s preferences of services; and e) interventions to break the link between vulnerabilities, particularly- modern day slavery and homelessness. Effective partnerships across diverse stakeholder groups were deemed to be imperative in developing, testing and implementing novel interventions.

**Conclusions:**

Maximising access to services, prevention of early deaths linked to drugs and alcohol, and identifying effective and ineffective policies and programmes were identified as priority research areas in relation to healthcare of PEH. The outcomes of this discussion will enable design and conduct of interdisciplinary research programmes to address the syndemics of homelessness and linked adverse health outcomes. Priorities identified here are likely to be applicable internationally.

## Background

United Kingdom (UK) is facing an increasing burden of homelessness and associated inequalities. There are an estimated 300,000 persons known to be homeless in England alone including over 4000 persons sleeping rough (i.e. street dwelling) on any given night [1]. Since 2013, the number of rough sleepers in urban areas such as London has risen two fold [1]. Welfare reforms introduced since the 2008 financial crisis, lack of adequate social housing and access to substance misuse and mental health services have often been linked to the upward trend [2, 3].

Homelessness and adverse health outcomes, particularly substance misuse and severe mental health are closely interlinked. For example, 40% of all deaths amongst persons experiencing homelessness (PEH) in England and Wales in 2018 were contributed by drug poisoning [4]. A recent study conducted with over 900 persons registered with a specialist homeless service in England showed prevalence rates of severe mental health problems, alcohol dependence, substance misuse and infectious hepatitis C were 7, 15, 3 and 9 times the rate of the general population respectively [5].

A syndemic correlation refers to ‘two or more afflictions, interacting synergistically, contributing to excess burden of disease in a population’ [6, 7]’ A syndemic correlation exists between inequality and associated morbidities amongst PEH. Syndemics pertain to the social determinants of health and provide a framework for health inequality to be understood in the context of both physiological and psycho-sociological dimensions, emphasising the need for interdisciplinary and cross-sector collaboration to address multiple complex needs.

Poor access to health and social care services are often the cause and consequences of homelessness. PEH experience multiple barriers to accessing services including lack of knowledge, as well as the physical and mental capacity to prioritise healthcare and navigate services [8]. Perceived stigma and discrimination can deter PEH from using mainstream services [8, 9].

Recent policy interventions within the UK aim to mitigate homelessness and adverse health outcomes. For example, in England, rough sleeping strategy was published in 2018 [10] which aimed to eliminate homelessness by 2027 by increasing bed spaces in city council accommodations, increasing access to substance misuse and mental health treatment. ‘Housing first’ which aims to provide ‘a stable, independent home and intensive personalised support and case management to PEH with multiple and complex needs’ was one of the key interventions to support this strategy [11]. The Scottish Government’s abolition of the priority needs assessment when offering accommodation to PEH, entitling anyone finding themselves homelessness to settled accommodation is another example [12]. Other policies include the Homelessness Reduction Act in England mandating hospitals to identify and refer PEH to accommodation providers [13].

Despite the policy initiatives, severe multiple disadvantages still exist amongst PEH and these have not improved over time. For example, as of 2018, average age of death of PEH in England is 41 for males and 43 for females [4]. Systematic review of international literature suggests under-diagnoses, and under-treatment of health conditions [14]. Lack of services that effectively meet the needs of this population are amongst the contributory factors [15, 16], underscoring the need for development, evaluation and implementation of novel interventions to address health inequalities experienced by PEH.

Patient and public involvement (PPI) in research allows effective interventions to be identified by drawing on the expertise and knowledge of patients and partners [17]. Stakeholder engagement is an essential component of PPI. This paper aims to present outcomes of a national stakeholder engagement event that was conducted to discuss research priorities in healthcare of PEH in UK. The outcomes of such discussion would enable the development of interdisciplinary research programmes to address the syndemics of multiple complex needs and homelessness. This stakeholder engagement event formed part of a wider research programme funded by Public Health England in relation to needs assessment around healthcare of PEH.

## Methods

A national stakeholder event was organised in Birmingham, United Kingdom, hosted by the University of Birmingham. This workshop aimed to engage diverse stakeholders from a variety of backgrounds. This study was conducted as a public involvement event as per the PPI principles [17]. All participants of the workshop verbally consented and offered no objections to the summary of the discussion to be published without identifying any quote to a particular attendant or speaker.

Twenty-six participant invitations were sent via email by the researchers (VP and PKJ) to a variety of health and social care sectors across England of which 20 attended. Participants included: clinical practitioners including hepatologists; substance misuse nurse; clinical service leads; anti-slavery network leads; public health practitioners including representatives from Public Health England; local authority representatives; homelessness charities; drugs and alcohol services; and academic researchers in the areas of public health, dental sciences, pharmacy and social work. Contacts were identified using researcher acquaintance and expert search through Public Health England regional offices.

The one-day workshop included keynote presentations from invited speakers, followed by participant-led discussion. Invited speakers included the Medical Director of Pathway, Faculty for Homeless and Inclusion Health who discussed the implementation and outcomes of multimodal interventions in primary and secondary health care for PEH, and a pharmacist who led a street outreach intervention for PEH using pharmacist independent prescribers, research facilitators highlighting research funding opportunities, and a representative from a metropolitan taskforce on homelessness.

### Data collection and analysis

The moderators from the study team with experiences in health services research, public health, clinical pharmacy practice and research, facilitated participant-led discussions. Participants were asked to consider: priority research areas; key questions that needed to be answered through research; and relevant outcomes and implications that such research could offer. Participants were asked to make notes during the individual group discussions and the workshop facilitators made their own notes. Notes were then used to identify priority research areas and produce associated narratives. The identified research areas alongside research questions, relevant methodology for research, study outcomes and potential study implications were then tabulated using a data extraction form. Data saturation was not intended and qualitative data analytical methods were not used given the public involvement nature of the work.

## Results

As presented in Table 1, a total of five research priority areas were identified. These are described in depth in this section. The research areas, associated research questions, relevant methodology, study outcomes and potential practice implications are also listed (Table 1).

**Table 1:** Research priorities, questions, relevant methodology, study outcomes and potential implications to practice

Priority Research areas	Research questions	Methodology	Study outcomes	Potential study implications
Improving access to services	-What barriers do PEH face in accessing primary healthcare services?	-Qualitative study, surveys with patients, healthcare professionals and social workers	-Barriers and enablers of access to healthcare	-Widening access to mainstream health services and specialist services aimed at PEH by addressing the barriers
	-How can knowledge and awareness of healthcare staff be improved in relation to rights access to services by PEH?	-Interventional methodology through training, education and distribution of ‘my rights of access to healthcare’ cards to frontline healthcare staff	-Knowledge and awareness of healthcare staff around rights of PEH to access services -Number of PEH with access to primary healthcare services	-Greater inclusion of PEH in mainstream healthcare, use of specialist homeless healthcare services by patients where needed
	-How can we understand the impact of local and national policy interventions such as the Homelessness Reduction Act, Housing First and health charging guidance?	-A time trend analysis investigating homelessness and adverse health outcomes prior to and after the introduction of relevant policies	-Homelessness in numbers, mortality in PEH, average age of deaths of PEH, number of PEH effectively housed, episodes of repeat cycle of homelessness per person	-Identifying effective and ineffective health and social care policies
Interventions to prevent drug and alcohol-related deaths	-How could substance misuse services be extended to the population in need?	-Undertaking pilot provision of existing substance misuse services through new avenues such as provision through outreach and specialist homeless healthcare services	-Number of affected persons who access services, number of drugs and alcohol-related deaths	-Maximising access to substance misuse services, reducing associated mortality
	-What unique barriers people with dual diagnoses of substance misuse and mental health face in accessing services?	-Qualitative study, surveys and case study with patients	-Barriers and enablers for accessing services by patients with dual diagnoses of mental health and substance misuse	-Enhanced provision of services to patients with dual diagnoses, addressing the link between dual diagnoses and homelessness
	-How can naloxone services be made more widely available?	-Qualitative study and surveys with patients, healthcare professionals and stakeholders	-Barriers to provision of naloxone services through a variety of settings such as through community pharmacy and outreach services	-Enhanced provision of services by addressing the barriers and facilitating the enablers, prevention of opioid-related deaths
Improving existing services through quality improvement and better integration, operationalising current policy drivers and best practices	-How can we improve patient engagement with services by redesigning patient pathways?	-Quality improvement methodology	-Number of people accessing primary healthcare, substance misuse and mental health services	-Enhanced access to services by PEH, preventing the use of emergency departments and improving health outcomes
	-How effective are novel services such as social prescribing?	-Interventional methodology in new geographical areas, observational data collection in areas where such services already exist	-Number of people utilising social prescribing services, number of people being referred to appropriate services, health outcomes in relation to the clinical area of focus	-Greater engagement of patients with existing and new services, greater liaison between service providers
	-How can we minimise fragmentation of care for PEH?	-Qualitative study with patients, healthcare professionals and wider stakeholders -Establishing and evaluating referral pathways through interventional methodology -Quality improvement methodology offering relevant services for PEH under one roof	-Barriers to minimising fragmentation of care -Number of affected patients who use services, associated health outcomes	-Enhanced access to services and improved health outcomes -Minimising delays in communication and transfer of information in relation to care of PEH
	-How can clinical and communication skills of healthcare professionals be improved in relation to specific needs of PEH?	-Qualitative study with patients and healthcare professionals to identify current gaps in knowledge and skills of healthcare professionals -Interventional methodology to improve knowledge and skills of healthcare professionals in relation to care of PEH	-Communication skills of healthcare professionals -Clinical outcomes in relation to management of multi-morbidity, prescribing in acute or long-term conditions, alcohol and substance misuse, mental health	-Greater engagement of patients with mainstream and specialist homeless healthcare services -Greater rapport of PEH with healthcare professionals - improvement in clinical and communication skills of healthcare providers in relation to specific needs of PEH
Research to identify PEH’s preferences around service delivery	-What do PEH prefer in relation to addressing their unmet healthcare needs?	- Qualitative study with PEH to identify unmet needs -Patient and public involvement to engage service users in services design and evaluation -Using existing healthcare utilisation data (primary, secondary and emergency healthcare) to identify unmet needs	-Unmet healthcare needs of PEH -Patient and public involvement in research, policy and practice	-Identifying services to meet the needs of PEH
Reinforcing the link between vulnerabilities - modern day slavery and homelessness	-Is there a link between modern day slavery and homelessness?	-Ethnography with affected persons to understand any links that exist	-Identifying how modern slavery and homelessness are inter-related	-Preventing homelessness and modern day slavery by early interventions and referrals, preventing adverse health outcomes

*PEH* Persons experiencing homelessness

### a) Interventions to improve access to services

Participants discussed the need to improve access to services for PEH. A focus on system reforms for prevention actions was highlighted and the importance of moving away from crisis management as the main strategy to address homelessness. An urgent need to intervene and address barriers that prevent homeless populations registering and accessing primary healthcare services was identified. They described interventions focused on primary care frontline staff such as ‘All Our Health learning resources’ [18] that emphasised the legal rights of PEH to register and access primary health services, which could improve the knowledge of staff and promote inclusivity. The impact of recent ‘health charging guidance’ for individuals who have ‘no recourse to public funds’, particularly foreign immigrants from lower socioeconomic backgrounds, was deemed by the participants to adversely affect health and contribute to homelessness. Participants commented that poor access to primary healthcare services impact on morbidity, mortality and increased utilisation of emergency health services. Longitudinal studies were needed to identify effective and ineffective policy interventions. Novel interventions such as non-medical prescriber-led outreach clinics were discussed.

Participants described that the commissioning of initial crisis management services e.g. provision of a safe warm place to eat and sleep should be followed by services to support long term health and well-being. The Housing First Initiative in the UK which aims to provide ‘a stable, independent home and intensive personalised support and case management to PEH with multiple and complex needs’ [11] was identified as an example of an effective model of intervention. Participants mentioned that some PEH do not engage with mainstream health and social care services or are unable to register to health services due to the lack of permanent residence, or de-prioritisation of health seeking behaviour. Such non-engagement was also related to ill mental health resulting in cognitive and emotional barriers. Outreach and mobile services hence would provide alternative access. The need to undertake an interdisciplinary, multi-agency approach, strengthening of existing relationships and working with a wider range of partners such as the anti-slavery network were identified. Examples mentioned including ‘doctors of the world’, safe surgeries, and ‘docs not cops’ initiatives being rolled out in various geographies of England.

### b) Interventions to prevent drug and alcohol related deaths

Increasing prevalence of drug and alcohol misuse was identified by participants to be a key area that needed to be addressed given their syndemic association with higher mortality rates. Participants described the need to identify mechanisms by which preventative and rehabilitation services can be extended to the affected population, and the methods that would allow effective identification and treatment of patients with co-existing substance misuse and mental health. Health services needed to reach out to the patients who did not access existing services. Participants also emphasised the need to promote existing harm reduction strategies such as naloxone services, detoxification and opioid replacement therapy. The use of nasal naloxone services being piloted to reduce opiate related deaths was mentioned as an example [19]. Consideration of causes and outcomes of addiction were deemed key to the development of interventions.

### c) Improving existing services through quality improvement and better integration

Participants discussed the need to integrate existing services and redesign patient pathways. They discussed that a single and isolated intervention without adequate referral pathways and plans for seamless care often do not offer favourable outcomes. Social prescribing [20], a model for local agencies to refer people to a link worker or directly to a range of local, non-clinical services was recognised areas as an innovative model. Link workers were deemed to connect people to community groups and statutory services for practical and emotional support. There was recognition of the need to include wider services including community pharmacy in social prescribing activities and promotion of self-care in this population [21].

Participants discussed that services are often fragmented for patients with multi-morbidity. Through effective integration; current services could fulfil many unmet needs. The Homelessness reduction act in England [13] was described as a positive policy intervention to promote effective cross-sector working. Participants expressed the need to improve specialist skills amongst practitioners working with homeless populations e.g. prescribing in acute or long-term conditions, alcohol and substance misuse, mental health. Effective communication skills and resilience in addition to speciality-specific skills were also deemed necessary. Reforming training and education of healthcare professionals including identifying barriers for change in adopting new practices was deemed imperative in implementing and sustaining new services [22, 23]. Follow up care was deemed to be difficult due to itinerant and chaotic lifestyles of PEH, resulting in no single care record being fully complete or accurate. Delays in communication and transfer of information often led to loss of contact with vulnerable individuals.

### d) Research to identify patient/client preferences

Participants deemed that improving access and outcomes of care requires consideration of patient preference in terms of where and how the service is delivered and by whom. They identified that research around patient preferences in relation to service access and use should be investigated through exploratory approaches using qualitative methodology. Engaging service users in service developments and redesign was imperative. Turning existing datasets into intelligence was key to identifying unmet needs.

### e) Investigating the link between modern day slavery and homelessness

Modern slavery relates to ‘trafficking of people, forced labour, servitude and slavery’ [24]. Participants mentioned that researching the link between homelessness and modern day slavery was important given the current lack of data and metrics to identify the link. Participants described the need to improve effective coding of suspected cases of modern day slavery when persons present for services. Effective partnership amongst government departments, National Health Service (NHS), public health services and anti-slavery networks was deemed imperative. National referral mechanisms needed to be established. Participants suggested the need to increase opportunistic signposting or referral to services during routine contacts with health, social care and third sector agencies. Effective coding, data collection and analysis to identify numbers of PEH who are susceptible or have already been victims of exploitation should inform the design/implementation of prevention and intervention programmes.

### Limitations

This public involvement session was undertaken in the UK with a limited number of participants from stakeholder groups. While participants represented a wide variety of expertise and disciplines, this event may not have been able to capture all research priority areas. Our study also did not involve PEH participation because this event mainly aimed to draw on the voice of the stakeholder groups involved in the care of PEH. However, the researchers drew their prior experiences of working with PEH when informing the discussion plans for this event [25–28]. Identifying priority areas of research in the future will require consensus and nominal group techniques [29] for participants to agree on the agenda and future priorities. The findings of this study can be expanded upon to undertake research using such rigorous methodology in the future.

## Conclusions

This public engagement event involving diverse stakeholders was able to identify key areas of consideration for future research around healthcare for PEH. Multi-agency and multi-partner work is required to support data collection to set up new services and drive improvements in existing services. Development, implementation and evaluation of novel interventions to improve access and include the experiences of service users and health outcomes requires interdisciplinary collaboration and health services research expertise. Such interventions may include services to maximise the inclusion of PEH in mainstream primary healthcare services, promoting the use of outreach services for preventative and interventional healthcare, preventing repeat homelessness by addressing substance misuse and severe mental health issues.

There is a need to involve and engage patients in identifying research priorities and to utilise the expertise of wider health professionals including allied health professionals, non-medical prescribers and the voluntary sectors. While this stakeholder discussion was undertaken in the UK with a limited number of stakeholders, the outcome of this stakeholder engagement event can be relevant for other nations with similar socioeconomic and demographic characteristics.

## Data Availability

All data generated or analysed during this study are included in this published article.

## Abbreviations

NHS: National Health Service
UK: United Kingdom
